# The Back Pain Consortium (BACPAC) Research Program: Structure, Research Priorities, and Methods

**DOI:** 10.1093/pm/pnac202

**Published:** 2023-01-09

**Authors:** Matthew C Mauck, Jeffrey Lotz, Matthew A Psioda, Timothy S Carey, Daniel J Clauw, Sharmila Majumdar, William S Marras, Nam Vo, Ayleen Aylward, Anna Hoffmeyer, Patricia Zheng, Anastasia Ivanova, Micah McCumber, Christiane Carson, Kevin J Anstrom, Anton E Bowden, Diane Dalton, Leslie Derr, Jonathan Dufour, Aaron J Fields, Julie Fritz, Afton L Hassett, Steven E Harte, Trisha F Hue, Roland Krug, Marco L Loggia, Prasath Mageswaran, Samuel A McLean, Ulrike H Mitchell, Conor O’Neill, Valentina Pedoia, David Adam Quirk, Daniel I Rhon, Viola Rieke, Lubdha Shah, Gwendolyn Sowa, Brennan Spiegel, Ajay D Wasan, Hsiao-Ying (Monica) Wey, Lisa LaVange

**Affiliations:** University of North Carolina at Chapel Hill, Chapel Hill, NC, United States; University of California San Francisco (UCSF), San Fransisco, CA, United States; University of North Carolina at Chapel Hill, Chapel Hill, NC, United States; University of North Carolina at Chapel Hill, Chapel Hill, NC, United States; University of Michigan (U Mich), Ann Arbor, MI, United States; University of California San Francisco (UCSF), San Fransisco, CA, United States; Ohio State University (OSU), Columbus, OH, United States; University of Pittsburgh (U Pitt), Pittsburgh, PA, United States; University of North Carolina at Chapel Hill, Chapel Hill, NC, United States; University of North Carolina at Chapel Hill, Chapel Hill, NC, United States; University of California San Francisco (UCSF), San Fransisco, CA, United States; University of North Carolina at Chapel Hill, Chapel Hill, NC, United States; University of North Carolina at Chapel Hill, Chapel Hill, NC, United States; University of North Carolina at Chapel Hill, Chapel Hill, NC, United States; University of North Carolina at Chapel Hill, Chapel Hill, NC, United States; Brigham Young University (BYU), Proto, UT, United States; Boston University, Boston, MA, United States; National Institutes of Health (NIH), Bethesda, MA, United States; Ohio State University (OSU), Columbus, OH, United States; University of California San Francisco (UCSF), San Fransisco, CA, United States; University of Utah, Salt Lake City, UT, United States; University of Michigan (U Mich), Ann Arbor, MI, United States; University of Michigan (U Mich), Ann Arbor, MI, United States; University of California San Francisco (UCSF), San Fransisco, CA, United States; University of California San Francisco (UCSF), San Fransisco, CA, United States; Massachusetts General Hospital, Harvard Medical School, Boston, MA, United States; Ohio State University (OSU), Columbus, OH, United States; University of North Carolina at Chapel Hill, Chapel Hill, NC, United States; Brigham Young University (BYU), Proto, UT, United States; University of California San Francisco (UCSF), San Fransisco, CA, United States; University of California San Francisco (UCSF), San Fransisco, CA, United States; Boston University, Boston, MA, United States; Uniformed Services University of the Health Sciences, Bethesda, MD, United States; University of Utah, Salt Lake City, UT, United States; University of Utah, Salt Lake City, UT, United States; University of Pittsburgh (U Pitt), Pittsburgh, PA, United States; Cedars-Sinai Comprehensive Transplant Center (CTC), Los Angeles, CA, United States; University of Pittsburgh (U Pitt), Pittsburgh, PA, United States; Massachusetts General Hospital, Harvard Medical School, Boston, MA, United States; University of North Carolina at Chapel Hill, Chapel Hill, NC, United States

**Keywords:** Chronic low back pain (cLBP), BACPAC Research Consortium, Harmonization, Back Pain, HEAL, SMART, clinical trials, chronic disease, chronic pain, low back pain

## Abstract

In 2019, the National Health Interview survey found that nearly 59% of adults reported pain some, most, or every day in the past 3 months, with 39% reporting back pain, making back pain the most prevalent source of pain, and a significant issue among adults. Often, identifying a direct, treatable cause for back pain is challenging, especially as it is often attributed to complex, multifaceted issues involving biological, psychological, and social components. Due to the difficulty in treating the true cause of chronic low back pain (cLBP), an over-reliance on opioid pain medications among cLBP patients has developed, which is associated with increased prevalence of opioid use disorder and increased risk of death. To combat the rise of opioid-related deaths, the National Institutes of Health (NIH) initiated the Helping to End Addiction Long-Term^SM^ (HEAL) initiative, whose goal is to address the causes and treatment of opioid use disorder while also seeking to better understand, diagnose, and treat chronic pain. The NIH Back Pain Consortium (BACPAC) Research Program, a network of 14 funded entities, was launched as a part of the HEAL initiative to help address limitations surrounding the diagnosis and treatment of cLBP. This paper provides an overview of the BACPAC research program’s goals and overall structure, and describes the harmonization efforts across the consortium, define its research agenda, and develop a collaborative project which utilizes the strengths of the network. The purpose of this paper is to serve as a blueprint for other consortia tasked with the advancement of pain related science.

## Introduction

The National Institutes of Health (NIH) Task Force on research standards for chronic low back pain (cLBP) defines cLBP as pain persisting for at least three months and occurring on at least half the days in the past 6 months [1]. Key findings of the 2019 National Health Interview survey show that of the nearly 59% of adults reporting pain *some*, *most*, or *every* day in the past 3 months, 39% reported back pain, making it the most prevalent source of pain [2]. Estimates of health care costs related to cLBP are variable, but some total cost estimates are greater than $100 billion per year [3]. Despite significant investments in basic research and novel therapies, rates of cLBP continue to rise worldwide. Efforts to address this widespread issue are confounded by the fact that, for most people with cLBP, identifying a clear cause of the pain is extremely challenging, largely because back pain is the symptom of a complex, multifaceted disorder, where biological, psychological, and social factors interact to affect the onset and trajectory of pain. As a result, there is no widely accepted standard for back pain diagnoses, and without a precision medicine approach to treating the condition, there exists a dependence on trial-and-error approaches that often fail to fully alleviate the pain or improve function and simply add to the rising cost of care. One consistent observation across cLBP clinical studies is that some patients respond well to any given treatment, while others do not respond at all. This leads to the current state in which there are a wide range of available interventions, each with varying efficacy in different subsets of individuals, but little understanding regarding who will respond to a given intervention.

This unpredictability of treatment benefit has contributed to an overreliance on opioid-based pain management. In the United States, cLBP has become the most common, non-cancer reason for opioid prescriptions, causing significant patient harms including opioid use disorder and increased risk of death [4]. In response to the rising rates of opioid-related deaths, the National Institutes of Health (NIH) launched the Helping to End Addiction Long-term^SM^ (HEAL) Initiative. This initiative was implemented to address the causes and treatment of opioid use disorder, while also focusing on understanding how to better diagnose and treat chronic pain. One component of the NIH HEAL Initiative is the NIH Back Pain Consortium (BACPAC) Research Program, a patient-centric, translational research program administered by the National Institute of Arthritis and Musculoskeletal and Skin Diseases (NIAMS). BACPAC is addressing the cLBP challenge by developing and testing a variety of novel approaches that jointly enable comprehensive clinical assessment and optimal treatment planning. The multi-component research program consists of both cohort studies and clinical trials designed to identify phenotypic markers and better understand the root mechanisms of cLBP, examine why different patient populations respond better to certain treatments, and test innovative non-opioid approaches to treating cLBP. This overview paper is intended to provide a high-level description of the BACPAC Research Program and its overarching goals, structure, and component projects as a model for an integrated NIH consortium designed to address a prevalent and expensive health challenge. Our hope is that by describing the BACPAC Research Program, this article may serve as a blueprint for developing and implementing other consortia to advance multidisciplinary science focused on the most common and impactful conditions.

## Network structure

To support collaborative research and cross-discipline learning, the BACPAC Research Program commenced in 2019 and comprises a number of complementary research components. These components work together to achieve the Consortium objectives of extensively phenotyping patients with cLBP, developing an integrated model of cLBP, producing new and improved diagnostic and treatment algorithms, and testing new therapies in clinical trials. The four research components are:

1. Data Integration, Algorithm Development, and Operations Management Center (DAC)
2. Interdisciplinary Mechanistic Research Centers (MRCs)
3. Technology Research Sites (Tech Sites)
4. Clinical Trial Centers (CTCs—Two phase 2 trials and one phase 3 trial)

Each research component reflects a diverse portfolio of research activities requiring collaboration, cooperation, and extensive data and resource sharing.

The DAC provides operational oversight to the Consortium through its coordinating center activities and is the architect of the BACPAC Data Portal, a data warehouse and data analytics platform developed to house and manage system-level analyses of consortium-wide data. The DAC also serves as the data and clinical coordinating center (DCC/CCC) for the BACPAC initiated Consortium-wide Sequential Multiple Assignment Randomized Trial (SMART), henceforth referred to as the Biomarkers for Evaluating Spine Treatments Trial (BEST). BEST is discussed in detail in the section “consortium wide collaborative research” of this article.

The three MRCs conduct translational research to characterize cLBP mechanisms and develop phenotyping algorithms in clinical cohorts. The research programs conducted by the MRCs include two large longitudinal observational studies that follow one or more cLBP cohorts and one SMART trial. The specific details of the MRC research programs are described in three separate papers within this special issue.

The seven Tech Sites conduct technology development and deployment to support greater exploration of the links between structural and cellular abnormalities and patient-reported symptoms and function. Tech Site research programs are focused on the adaptation of existing technology as well as the development of new technology to phenotype cLBP patients, identify biomarkers of cLBP, and potentially treat cLBP. The objectives of each Tech Site are described in the section “novel tools and emerging technology” of this article. The specific details of four of the Tech sites are described in separate papers within this special issue.

The CTCs are conducting two phase 2 and one phase 3 clinical trials to assess the safety and efficacy of novel, non-opioid therapies for cLBP. Research across the three trials varies and includes the use of therapeutic virtual reality (VR), anti-depressants and fear-avoidance therapy, and a comprehensive post-surgical pain management approach to reduce opioid use for cLBP. The specific details of two of the clinical trials are described in separate papers within this special issue. Details on the VR trial, conducted at Cedars-Sinai, have been published elsewhere [5].

**Figure 1.**
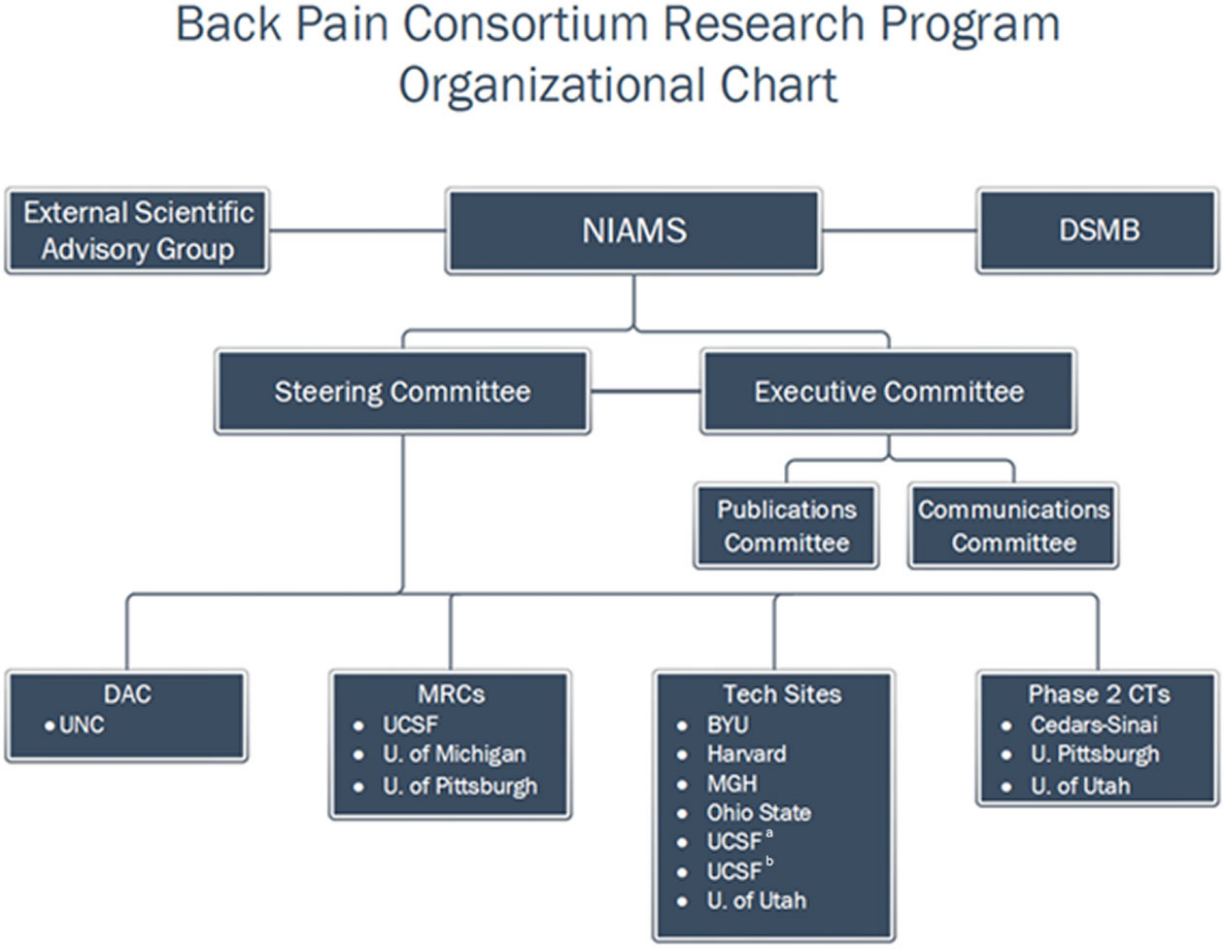
CTs = clinical trial centers; DSMB = data and safety monitoring board; DAC = data integration, algorithm development and operations management center; MRCs = interdisciplinary mechanistic research centers; NIAMS = national institute of arthritis and musculoskeletal and skin diseases.

The BACPAC Governance structure is depicted in Figure 1. The Steering Committee (SC) is the primary governing body and is responsible for research agenda development, prioritization and allocation of funds for pilot and ancillary studies, approval of the BEST design, and BEST budget recommendations to NIAMS. The SC comprises the primary investigators from each of the 14 BACPAC research components and NIH representatives. SC members are:

- University of California San Francisco (UCSF) MRC (Jeffrey Lotz, PhD),
- University of Michigan (U Mich) MRC (Daniel Clauw, MD/Afton Hassett, PsyD),
- University of Pittsburgh (U Pitt) MRC (Gwendolyn Sowa, MD, PhD/Nam Vo, PhD),
- Brigham Young University (BYU) Tech Site (Anton Bowden, PhD, PE),
- Harvard University Tech Site (Conor Walsh, PhD),
- Massachusetts General Hospital (MGH) Tech Site (Hsiao-Ying (Monica) Wey, PhD),
- Ohio State University (OSU) Tech Site (William Marras, PhD, CPE),
- UCSF Tech Site (Aaron Fields, PhD/Roland Krug, PhD)^a^,
- UCSF Tech Site (Sharmila Majumdar, PhD)^b^,
- University of Utah Tech Site (Viola Rieke, PhD/Lubdha Shah, MD),
- Cedars-Sinai CTC (Brennan Spiegel, MD, MSHS),
- University of Pittsburgh CTC (Ajay Wasan, MD, MSc),
- University of Utah CTC (Julie Fritz, PT, PhD/Daniel Rhon, DPT, DSc, PhD), and
- University of North Carolina at Chapel Hill Data Integration, Algorithm Development and Operations Management Center (DAC) (Kevin Anstrom, PhD/Anastasia Ivanova, PhD/Lisa LaVange, PhD).

The Executive Committee (EC) comprises representatives from the DAC, NIAMS, MRCs, and Tech Sites. The EC sets the SC agenda and oversees Consortium-wide activities, in particular, the work of committees such as the Data Access and Publications Committee and Clinical Management Committee, whose activities impact the entire Consortium. The EC also monitors the progress of working groups established by the SC to focus on key components of the BACPAC Research Program.

Early in the project period, the BACPAC SC established ten working groups and four committees, in addition to the EC and SC, to facilitate achievement of the Consortium research goals. Working groups and committees were populated with members from the DAC, MRCs, Tech Sites, and CTCs who then developed charters, timelines, and a list of milestones and deliverables for each of the respective groups. The specific working groups and committees instantiated by the BACPAC SC and a brief statement of their deliverables are described in Table 1.

**Table 1.** BACPAC working groups and committees.

Working Group (WG) or Committee	Deliverable(s) and Charges
Biobehavioral Research WG	Recommendations for optional psychosocial measures to be collected in addition to minimum data set Recommendations for best practices for patient assessments Recommendations for a quantitative sensory testing (QST) minimum data set
Biomechanics and Physical Function WG	Recommendations for patient-specific biomechanical modeling approaches to be utilized to phenotype mechanical sources of chronic low back pain
Biospecimen Collection and Processing WG	Gap analysis on cross-study specimen collection, storage, processing and distribution Recommendations for additional consortium-level specimen collection/analysis Recommendations for modifications, refinements, and/or additions to the EPPIC-Net Laboratory manual SOPs for biospecimen collection, storage, processing, and distribution Training videos and corresponding materials for biospecimen collection, storage, processing, and distribution
Brain Imaging Studies WG	Harmonized network T1, T2, resting state protocols Recommendations for site-specific protocols Harmonization of network scanners
Data Analysis Methods WG	Collection, peer review, and approval of statistical analysis plans from MRCs and Tech Sites Recommendations for best practices using analytical methods
Data Sharing, Management, and Standards WG	SOPs for data transfer of: – Data flow within the Consortium – BACPAC minimum data set and additional broadly collected PRO measures – Biomechanical, physical function, and QST data – Omics data including genomics, epigenomics, proteomics, and metabolomics – Imaging data Data standards definitions for: – BACPAC minimum data set and additional broadly collected PRO measures – Physical function and QST data – Biomechanical data
Minimum Data Set and Outcome Measures WG	BACPAC definition of cLBP BACPAC required demographic measures BACPAC required outcome measures
Spine Imaging Studies WG	SOP for spine MRIs Proposal for standard scoring of spine MRIs
Systems Biology and Bioinformatics WG	SOPs for: – Batch Harmonization – Clustering – Differential Gene Expression – Genotype Calling – Predictive Modeling – Network Analysis
Theoretical Models for cLBP WG	Develop an integrative theoretical model to explain how potential risk factors, and their interactions, contribute to the experience of chronic low back pain Develop sub-models to expand on the overall model, and definitions of related model domains Generate mappings between model domains and clinical data elements being collected through the various BACPAC clinical studies Collect literature to support the model Develop list of hypotheses from BACPAC research projects and use the model to identify gaps Acquire existing clinical data sets from inside and outside BACPAC as a resource for model refinement Host research projects using existing clinical data to test and refine the theoretical model
Clinical Management Committee	Support activities of the Minimum Dataset and Outcome Measures Working Group Serve as the clinical expert group to support DAC clinical operations for funded research activities
Communications Committee	Develop public-facing materials for BACPAC Develop public-facing materials for BEST Coordinate BACPAC webinars
Data Access and Publications Committee	BACPAC Data Access and Publications Policy

PRO = patient reported outcome; SOP = standard operating procedures.

Success of the Consortium requires consensus on common data elements, protocols, and standards across the four research components comprising BACPAC. As such, the first year of the program focused on harmonization activities. The SC oversaw 10 working groups charged to develop clinical protocols, standard operating procedures, staff training plans, recruitment plans, electronic health record standardization, safety standards, and regulatory processes. Table 1 provides a list of the individual working groups and their specific deliverables and responsibilities.

These programmatic activities established a robust foundation on which to: (1) collect a rich data set for deep phenotyping (involving patient-reported outcomes and clinical, biomechanical, imaging, biospecimen, and genomic data); (2) develop mechanistic models to more precisely characterize the relationship between cLBP and physical function using a biopsychosocial approach; (3) validate novel phenotyping and/or interventional technologies for use in cLBP; and (4) evaluate several interventions in clinical trials. In addition to project-specific research activities, data generated by the Consortium will be harmonized and broadly shared to support integrative analyses across studies, enable cross-validation of project-specific findings, support the design of future studies (e.g., pilot studies), and bolster novel scientific research that complements activities already planned for the initially funded BACPAC projects.

The Consortium research program addresses fundamental questions about cLBP to improve the understanding of this condition and individual experiences, generate new information on possible connections between phenotypes and treatments, and identify novel pathways and targets for intervention. Three categories of Consortium research function synergistically to optimize translation of new scientific findings and technologies into better approaches for improving the lives of cLBP patients:

1. **Development of the BACPAC theoretical model for cLBP:** This model is based on the current state of knowledge in the scientific community regarding biological, biomechanical, psychosocial, and other processes (e.g., anatomical, psychological, etc.) that govern the manifestation of cLBP. Relationships implied by the theoretical model are interrogated using data generated by or available to the BACPAC with an aim to revise the model over the 5-year BACPAC Research Program to reflect a state-of-the-art model for cLBP.
2. **Development of novel hypotheses about possible interventional phenotypes for cLBP:** Expertise and resources available to the Consortium are leveraged to develop novel hypotheses about possible interventional phenotypes for cLBP (i.e., factors that are predictive of treatment effectiveness for well-defined patient subpopulations). Hypothesis generation is achieved in part by identifying treatments that target specific pathways implied by the BACPAC theoretical model. To the extent possible, these hypotheses are tested using the data generated by the BACPAC Research Program or using existing data sets obtained by BAPAC that could support these activities. The Consortium is also conducting selected pilot and ancillary studies designed to support elucidation of interventional phenotypes, with some preference given for studies using technology and approaches developed by BACPAC research sites testing (1) novel interventions or (2) novel approaches to phenotype patients. The selection and prioritization of research activities is informed by the scientific research gaps analysis conducted by the BACPAC.
3. **Design and conduct of a large-scale adaptive cLBP trial testing multiple interventions:** BEST utilizes a SMART design to address specific questions regarding interventional phenotypes that are not otherwise being addressed within BACPAC or elsewhere. The key study objective is to identify advanced phenotypes and optimal treatment strategies for patients who are categorized with those phenotypes. BEST launched in year 3 (2022).

## Novel tools and emerging technology

Given the complexity of cLBP, improvements in clinical care will likely require novel technologies. The BACPAC Tech Sites are focused on developing, validating, and deploying novel analytic tools, technologies and methods (TTM) to improve our understanding of cLBP mechanisms and thereby identify patient phenotypes in whom different mechanisms are the drivers of the pain. Tech Site advancements will be useful for sub-phenotyping of patients, prediction of treatment response, and contributing to algorithms for individualized treatment plans. The timeline for the Tech Sites includes a 1–2 year technique development (UH2) phase, followed by a 3–4 year implementation, validation and deployment phase (UH3). Some of the tools developed through these projects are deployed in BEST. The tools are scalable, and non- or minimally-invasive so that they may generalize to adoptable strategies. Together with the MRCs and the DAC, Tech Sites are exploring links between specific structural, dynamic, cellular or molecular abnormalities and specific patient-reported symptoms and function.

The multiple Tech Sites employ a mix of exploratory and focused research development projects that includes broad developments in imaging and biomechanics and therapeutics. The specific tools are described below.

### Imaging tools

#### PET/MRI of epigenetic dysregulation

Neuroepigenetic mechanisms have been linked to the development and maintenance of pain through preclinical models of inflammatory and neuropathic pain. One family of epigenetic enzymes, known as histone deacetylases (HDACs), are being considered as therapeutic targets due to the analgesic responses achieved through HDAC inhibitors. Inhibition of HDACs leads to symptom amelioration in experimental models of pain. There is, however, limited evidence about HDAC density concerning human pain across the entire brain. Significant research developments at MGH have demonstrated that a Positron Emission Tomography (PET) imaging agent, [^11^C]Martinostat, selectively binds to a subset of HDAC enzymes and has robust brain uptake and high specific binding. BACPAC takes the next step to develop [^11^C]Martinostat PET as a quantitative image biomarker for pain detection and diagnosis, with an ultimate goal of using [^11^C]Martinostat PET to monitor treatment responses. Using [^11^C]Martinostat PET/MR imaging in humans helps answer fundamental questions about chromatin-modifying enzymes in the living human brain. Importantly, using [^11^C]Martinostat to understand the alternation of HDAC expression in chronic pain patients enables validation of an epigenetic drug target, refines patient selection based on HDAC expression, and facilitates proof of mechanism/target engagement in developing novel analgesics. Initial proof-of-concept clinical validation studies are being conducted to evaluate if [^11^C]Martinostat PET is a sensitive biomarker to detect typical (axial) cLBP. The goal of further explorations is to establish the validity of using [^11^C]Martinostat PET to differentiate subtypes of pain by comparing [^11^C]Martinostat PET binding in axial cLBP patients with binding in cLBP patients with radiculopathy. Longitudinal studies in sub-acute LBP patients (sLBP) are then used to investigate whether there is a unique imaging signature that differentiates patients who convert to cLBP compared to those who recover from low back pain.

#### Quantitative MRI and deep learning

Quantitative magnetic resonance imaging (MRI) has been used to characterize disc, muscle, and nerves, and PET has been used to study bone turnover and facet disease, in subjects with cLBP. Often, lack of reproducibility, long imaging and analysis times, and lack of specificity and sensitivity are barriers to the utilization of quantitative imaging biomarkers. The research and tool development completed at the UCSF Tech Site (Majumdar) took the critical next step in the clinical translation of faster MRI of patients with lower back pain. Leveraging key technical advancements such as the development of machine learning, this UCSF Tech Site has developed deep-learning-based technologies for accelerated image reconstruction, tissue segmentation, and detection of spinal degeneration (such as disc Pfirrmann grade, Modic changes, stenosis, and facet degeneration) to facilitate automated, robust assessments of structure-function relationships between spine characteristics and neurocognitive pain response measured using brain MRI and patient reported outcomes. This UCSF Tech Site is focused on the validation and testing of these tools in building biomechanical models in collaboration with other BACPAC Tech Sites at Beth Israel Deaconess Medical Center and OSU. The success of PET/MRI-guided facet and nerve blocks in alleviating patient symptoms is leveraged in BACPAC, and the tools developed and validated are tested in two cohorts of subjects undergoing (i) PET/MRI-guided facet and nerve block and (ii) integrative mindfulness therapy, to ensure the characterization of baseline pain, and treatment response is comparable when using machine-learning-enabled imaging and standard methods. The goal is to incorporate the minimal risk methods into BEST.

#### MRI of endplate biomarkers

UCSF Tech Site (Fields/Krug) is further advancing the use of imaging in BACPAC by discovering clinically relevant biomarkers of endplate pathology, which includes focusing on novel imaging measures of endplate bone marrow lesion (BML) severity and cartilage endplate (CEP) fibrosis/damage; assessing interactions with paraspinal muscles; and identifying metrics that associate with pain, disability, and treatment response. Using existing legacy methods in conventional MRI while also developing new techniques based on advanced MRI, the team is refining methodologies for measuring imaging biomarkers of BML severity and CEP fibrosis. Studies will determine if including these new imaging biomarkers significantly improves predictive models of pain and disability compared to models with conventional MRI that are not sensitive to BML severity or CEP fibrosis. BACPAC research at this Tech Site also involves translational studies in patient cohorts undergoing treatments that may be influenced by BML severity and/or CEP fibrosis. The ultimate goal of these studies is to improve patient selection for nerve ablation therapy and intradiscal biologic therapy. Overall, the tools developed in at this Tech Site are designed to facilitate addressing the endplates' role in cLBP, identifying sub-phenotypes, discovering pain mechanisms, uncovering treatment targets, and selecting patients. The goal is to incorporate the minimal risk methods into BEST.

### Novel therapeutic tools

#### Focused ultrasound neuromodulation

Traditional interventional pain procedures for cLBP often lack long-term efficacy and are associated with procedural risks. To address this, the team at the University of Utah Tech Site is developing a focused ultrasound (FUS) cLBP therapy that is completely non-invasive and delivers spatially confined acoustic energy to the dorsal root ganglion [DRG] under magnetic resonance (MR) guidance. The central goal of this study is to demonstrate that DRG neuromodulation with FUS can decrease nerve conduction, and thereby be used to attenuate pain sensation. The project first establishes electrophysiologic normative data for detecting changes in pain measured by EEG and somatosensory-evoked potentials. Next, a large animal model is used to demonstrate efficacy of FUS neuromodulation of the DRG. To enable translation of these results to the clinic, an LBP-specific MR FUS device is designed and constructed for patients with back pain. This product development includes characterizing FUS sonications for DRG neuromodulation using regulatory standards, constructing an MRI radiofrequency coil and transducer mount to allow targeting of the DRG in humans, and evaluating the prototype for image and sonication quality. By demonstrating that neuromodulation with FUS can alter pain perception (by cortical monitoring and behavioral assessments), this research advances the ultimate goal of developing a completely non-invasive system to treat cLBP that includes real-time treatment adjustment based on the patient’s cortical response. The investigators expect that FUS will be a noninvasive modality to treat cLBP and has the potential to replace current invasive or systemically detrimental treatment modalities.

### Wearable sensors and skeletal biomechanics:

#### Robotic apparel

The Harvard University Tech Site is focused on developing robotic apparel for alleviating cLBP. The technology consists of a soft robotic exoskeleton designed to actively deliver supportive forces to the back and hip based on motion sensors. Using an active controller, this device adapts to an individual’s movement, delivering assistance specific to the direction, speed, and range of motion of a bending task. It is anticipated this adaptive assistance will reduce exertion across varied movement strategies, to thereby promote recovery over time as an individual recovers from cLBP. The technology is designed to prevent cLBP in individuals who are exposed to overexertion and supplement ergonomic training. However, robotic apparel can also provide a new tool to physical therapists and the clinical community to enhance rehabilitation programs by assisting in safe progression back to normal activity, enabling people to get back to activity sooner while encouraging adaptive movement strategies. This research is being conducted in a staged approach and is based on a human-in-the-loop development process that evaluates component and system functions through frequent human subject studies, where quantitative (robot, biomechanical and physiological) as well as qualitative (i.e., human factors) data are collected.

#### Printable nanocomposite sensor

The BYU Tech Site is developing a SPInal Nanosensor Environment (SPINE Sense System) to measure lumbar kinematics at various points along the spine with the objective of providing an objective, quantitative platform for diagnosis, monitoring, and follow-up assessment of cLBP. This effort employs a series of unique, inexpensive, screen-printable, elastomer-based nano-composite piezoresponsive sensors which are integrated into a SPINE Sense System to measure lumbar kinematics. The sensors themselves are screen-printed onto off-the-shelf athletic tape (e.g., KT Tape) and then attached to the skin of the back, changing resistance as a function of the 3D kinematic motion of each functional spinal unit. An attached microcontroller communicates these resistance changes via Bluetooth Low Energy to the subject’s smartphone. A machine-learning model integrated into the smartphone interprets the resistance changes to provide accurate, real-time, in vivo tracking of the 3D motion of the spine. This technology is expected to enable clinicians to have a window into objective measures of spinal kinematics during diagnosis, treatment, and post-treatment monitoring.

#### Wearable sensor for phenotyping

The OSU Tech Site is developing a novel Digital Health Platform that can collect, analyze and present novel quantitative measures of low back function by combining wearable motion sensor data with a holistic sample of biopsychosocial measures taken from patient-reported outcomes in one unified environment. Wearable sensors consist of inertial measurement units (IMUs) worn on harnesses placed around the back and hips. Collectively these sensors document the kinematic signature of a patient’s back motion in 3D. Patient-reported outcomes can be selected from among over 70 questionnaires included in the system platform. All data are collected in a cloud environment where they can be efficiently consolidated and analyzed from various collection sites located around the nation. Machine learning is used to assess the kinematic/biopsychosocial metrics. The utility of this system and the measures captured are being evaluated via a large validation study that aims to use patient phenotyping to predict treatment response probabilities and facilitate personalized medicine. Collectively, this effort has the potential to shift clinical practice paradigms, improve patient outcomes, enhance care efficiency, and reduce costs.

## Harmonization of data collection measures and protocols

### BACPAC minimum required data set for patient reported outcome measures

The BACPAC Research Program was charged with developing a minimum data set that would allow the Consortium to consistently characterize a large sample of patients across the 13 research sites with respect to their demographics, pain characteristics, and risk factors. To meet this charge, a Minimum Data Set and Outcome Measures Working Group (MDSWG) was convened.

Using initiative-required demographics and outcomes measures and the 2014 Report of the NIH Task Force on Research Standards for cLBP (1) as starting points, the MDSWG developed BACPAC-specific requirements for data collection across the Consortium, including a definition of cLBP, demographic questions, and a set of outcome measures to be assessed at baseline and follow-up visits. In addition to the NIH HEAL Initiative^SM^ demographic and outcome measures, questions on the relative severity of low back pain, as compared to other pain conditions, and household size, were included in the BACPAC minimum data set. Building further on the initiative data set, the BACPAC minimum data set adds PROMIS measures for pain interference, anxiety and depression and incorporates items to assess widespread pain, pain somatization, and current opioid use (Table 2). Additionally, the MDSWG established timepoints for data collection and made recommendations for non-essential measurement domains.

**Table 2.** 

	NIH HEAL Initiative Core	Additional BACPAC measures
Pain intensity	*Pain, Enjoyment of Life and General Activity scale (PEG)	Low-back pain specific pain intensity
Pain interference	*PEG	PROMIS-4 item Pain Interference
Physical function/QOL	PROMIS Physical Functioning Short Form 6 b	
Sleep	PROMIS Sleep Disturbance 6a + Sleep Duration Question	
Pain catastrophizing	Pain Catastrophizing Scale—Short Form 6	
Depression	PHQ-2	PROMIS-4 item Depression
Anxiety	GAD-2	PROMIS-4 item Anxiety
Global satisfaction with treatment	PGIC	
Substance use screener	TAPS 1	
Pain location		Radicular Pain Questions Adapted from NIH Research Task Force Minimum Dataset
Pain somatization		Abbreviated Pain Somatization Adapted from NIH Research Task Force Minimum Dataset
Widespread pain		Widespread Pain Inventory
cLBP Definition: pain duration and frequency		2 Items (low-back pain duration and frequency) from NIH Research Task Force Minimum Dataset
Opioid use		Single-Item Current Opioid Use
Demographics	Initiative-specific questions	BACPAC-specific questions

PROMIS = patient reported outcomes measurement information system; PHQ-2 = patient health questionnaire—2 item; GAD-2 = generalized anxiety disorder—2 item; PGIC = patient global impression of change; TAPS 1 = the Tobacco, Alcohol, Prescription medication and other Substance use tool.

The BACPAC minimum data set is required for any BACPAC research project involving longitudinal follow-up of cLBP patients. Outcome measures are assessed at baseline and at 3 months (± 2 weeks), with the exception of Patient Global Impression of Change (PGIC), which is assessed only at 3 months. The MDSWG recommended that individual projects choose additional measurement periods based on the aims of their projects.

### Harmonization of MRC studies

Further harmonization efforts for non-required domains were undertaken by the Clinical Management Committee (CMC). These efforts required balancing Consortium priorities, site priorities, site capacity, and patient burden across BACPAC, particularly for studies conducted by the MRCs. Key areas of harmonization include inclusion/exclusion criteria, the assessment of comorbidities and pain conditions, and the characterization of treatments received during observational studies.

### Inclusion/exclusion criteria

The CMC sought to harmonize the inclusion and exclusion criteria across studies conducted by the MRCs, which will together enroll up to 7,800 participants in in-person and online studies. Representatives from each of the MRCs shared their sites’ planned inclusion and exclusion criteria, and an iterative process followed during which the CMC identified areas of overlap and conflict among sites. As part of the collaborative process, MRCs adjusted data collection protocols where possible to facilitate later combined data analyses. Pregnancy, for example, is an exclusion criterion for only two of the MRCs, but the remaining MRC agreed to assess pregnancy status as well in order to support harmonization. The CMC also produced a written plan for how sites would collect data for inclusion/exclusion criteria that were not applicable across all MRCs.

### Comorbidities and chronic overlapping pain conditions

Comorbidities are relevant to characterizing the study sample and determining cLBP phenotypes, and, given the frequent co-occurrence of painful conditions for the cLBP population, the CMC agreed that non-cLBP chronic pain conditions were important comorbidities to harmonize as well. To this end, the CMC selected the Charlson Comorbidity Index (CCI) as a required measure for MRC studies and as a recommended measure for other sites (Tech Sites and Phase 2/3 CTs). Widely used in clinical practice and well validated [6–8], the CCI can be administered in person or assessed by querying Electronic Health Records (EHR) for relevant ICD-10 codes, a list of which the CMC defined for BACPAC using guidelines from Williams et al. [8]. To ensure a more comprehensive assessment of Covid-19 comorbidities, the CMC also added two questions (COVID diagnosis and hospitalization) to the assessment. As with the CCI, MRCs may assess the presence of overlapping pain conditions via EHR query using these ICD-10 codes or by patient self-report.

### Treatment characterization

Understanding the treatments that patients initiate, discontinue, or modify while enrolled in BACPAC’s observational studies strengthens the Consortium’s ability to conduct cross-study analyses that assess treatment effects and phenotypic variations in treatment effects and is useful in informing subsequent collaborative trials such as BEST. The CMC developed the Treatment Categories questionnaire to standardize collection of these data which can then be used to assess the type, intensity, duration, and effect of treatments (Supplemental Data Appendix 2).

### Additional data collection recommendations

The CMC worked closely with the Theoretical Model Working Group to ensure that BACPAC sites would collect data aligned with the BACPAC theoretical model of cLBP. Members of the CMC reviewed responses to a Consortium-wide comprehensive survey, in which sites outlined each questionnaire they intended to use. Responses were then mapped to the theoretical model elements to ensure that each would be covered with real-world data.

## Consortium wide collaborative research

### Collaborative data sharing and analysis

A highly unique and forward-thinking aspect of BACPAC, and the NIH HEAL Initiative^SM^ more broadly, is the goal of sharing data beyond independent research teams during the life of the studies in which the data are being collected. Whereas the initiative has put forth policies for broad data sharing with the scientific community at the time key study findings are disseminated by research teams, BACPAC has developed specific policies and infrastructure to support data sharing and collaborative analysis within the Consortium even more rapidly.

### Data transfer policy for collaborative data sharing

The Data Transfer Policy sets forth requirements for periodic transfer of cumulative data for many ongoing research studies to be securely housed in the DAC-hosted *BACPAC Data Portal*. Data files and associated metadata will adhere to standards developed by the Data Sharing, Standards, and Management Working Group to facilitate ease of use and downstream integration across studies. Once hosted on the BACPAC Data Portal, version-controlled data assets are then available for use for approved purposes by BACPAC researchers in accordance with the *Data Access and Publications Policy*.

### Data access and publications policy

The *Data Access and Publications (DAP) Policy* establishes a framework that facilitates access to the data stored on the BACPAC Data Portal, facilitates collaboration of BACPAC investigators on manuscripts, and provides record keeping for BACPAC’s published manuscripts, meeting abstracts, and presentations.

Investigators outside of the BACPAC Research Program may also request access to the BACPAC Data Portal for submission of data and/or to conduct analyses within the platform as a BACPAC *Affiliate*. Affiliate status requires the endorsement of an NIH representative or a BACPAC member institution PI and approval by the BACPAC’s EC. Once the DAP Committee has approved a request to use the BACPAC Data Portal, the requestor must establish a data use agreement with the DAC per University of North Carolina (UNC) institutional requirements and provide IRB approval/determination for their proposed research activities before the requestor is granted access.

### BACPAC data portal for collaborative analysis

The BACPAC Data Portal serves as the central repository for data generated by BACPAC projects or acquired from research studies conducted outside of the Consortium for purposes of integrative analyses. The BACPAC Data Portal is a secure, efficient, and easily accessible cloud-based system, built using Microsoft Azure, which serves as a comprehensive data warehouse with robust data asset search functionality. The Portal provides a high-performance computing environment based on scalable virtual machines (VMs) that are pre-configured to support both large and small computational needs. These VMs are provisioned with a comprehensive array of software tools (e.g., statistical analysis software such as R, SAS, and Stata). Researchers have the capability to export analysis results from the BACPAC Data Portal, but data are maintained securely through read-only controlled access.

### Patient engagement

BACPAC is, by design, a patient-centric research program, and significant effort has been placed on engaging patient and community stakeholders. UCSF and Cedars-Sinai have set up formal Patient Advisory Boards consisting of individual patients, representatives of chronic pain patient groups, and leaders of local centers for community engagement. The University of Michigan MRC, through a BACPAC supplemental grant, has established a patient advisory board specifically designed to support diversity, equity, and inclusion in the composition of study participants. A BACPAC Patient Board has been formed to guide the design, implementation and dissemination of findings from BEST. These patient and stakeholder boards contribute their expertise regarding individual and collective experiences of back pain treatment and back pain itself that are central to successful implementation of BACPAC research. Continued patient input on BACPAC studies will help researchers develop effective materials and language for the recruitment of patients from diverse ethnic, racial and socioeconomic groups and across the lifespan of cLBP, as well as ensure that studies incorporate culturally appropriate data collection measures. Patient involvement also helps to ensure that results will be useful and important to patient communities and responsive to the needs of individuals living with cLBP, and it fosters co-learning and community-building among patient, researcher and clinician groups. Finally, their guidance helps ensure eventual successful dissemination of findings by developing the appropriate plans and language for press releases and communications.

### Overview of the BEST design

Although a broad range of treatments for cLBP exists, current treatments do not adequately resolve the condition for most patients. Research into optimal treatment strategies for cLBP is challenging due to the diverse etiology of back pain, the varied phenotypes of cLBP patients, and the difficulty assessing the cause and contributors of pain, as well as the barriers to recovery. BEST is designed to address these shortfalls.

BEST (NCT05396014) is a multi-site, open label, sequential, multiple assignment randomized trial (SMART) to evaluate four interventions for cLBP: acceptance and commitment therapy, duloxetine, evidence-based exercise and manual therapy, and enhanced self-care (ESC).

The primary objective of BEST is to develop a precision medicine algorithm or dynamic treatment regime (DTR) based on individual phenotypes to treat cLBP patients. Currently, there are no precision medicine algorithms incorporating patient phenotypes to guide treatment decisions in cLBP.

Each participant will complete two 12-week treatment periods. Treatments are randomly assigned to participants at the start of the first treatment period. At the end of the first treatment period, PGIC and PEG scores will be assessed, and based on those reported outcomes, participants will either (1) maintain current treatment, (2) augment the current treatment with a randomly selected additional treatment, or (3) switch to a randomly selected new treatment. Participants reporting a PGIC score of 1–2 (defined as “Very Much or Much Improved” for BEST) will be randomized to strategy (1) (PEG <4) or strategy (2) (PEG >= 4). Participants reporting a PGIC score of 3–4 (defined as “Minimally Improved or No Change” for BEST) will be randomized between strategies (2) and (3). Participants reporting a PGIC score of 5–7 (defined as “Minimally, Much, or Very Much Worse” for BEST) will be randomized to strategy (3). Phenotypic assessments (PROs, biospecimen collection, biomechanical assessments, QST, and imaging) will be conducted at regular intervals during the two 12-week intervention periods, and it is this data that will inform precision medicine algorithms to support treatment optimization for future cLBP patients.

## Conclusion

BACPAC is an opportunity to advance our understanding of the mechanisms of cLBP through interdisciplinary, collaborative scientific investigation with the overarching aim of improving the care of individuals living with this debilitating condition. For the estimated 10%–20% of US adults who live with cLBP, the optimal treatment strategy is often not known, and there are no widely available clinical tools that can determine the best treatment plan for an individual experiencing cLBP. Guided by a broad research agenda and innovative theoretical model, all phases of the BACPAC Research Program are designed to generate multidimensional data to strengthen cLBP phenotyping, a key gap in cLBP research. Over the five project years, the Consortium is refining a theoretical model of cLBP, generating and testing novel hypotheses through analysis of both BACPAC data and data from ancillary studies, and conducting a large-scale clinical trial to identify optimum treatments based on patient phenotypes. Enhancing these efforts is the integration of novel phenotyping and interventional tools, which are being developed by BACPAC Tech Sites, validated, and ultimately incorporated into the Consortium’s collaborative clinical research efforts. The preparatory efforts completed to date and collaborative activities currently ongoing will result in tools that optimize non-opioid treatment of cLBP, including novel diagnostic and treatment technologies, a theoretical model that can be used by providers to aid clinical decision making, a high-quality data set for exploration by the broader biomedical research community, and algorithms to stratify patients into the optimal path of care.

Future research consortia of this magnitude could benefit from lessons learned in BACPAC. During the start-up phase of the project, we benefitted from our ability to integrate investigators and key project personnel across all the participating institutions and funding agencies for a kick-off meeting in November 2019 soon after the grants were awarded and, importantly, before the pandemic struck and rapidly diminished such opportunities. The speed with which committees and working groups were organized and subsequently finalized their work enabled several critical milestones associated with successful project start-up to be achieved, including identification of common data elements, initial framework on the theoretical model for cLBP, and a discussion of technical innovations that could inform the design of the BACPAC collaborative clinical trial. As work progresses along the many dimensions of the BACPAC program, we will accumulate additional, valuable lessons to inform future consortia with similar research goals and plan to share them through later publications.

## Supplementary Material

pnac202_Supplementary_Data
