# Supplementary material for: The Back Pain Consortium (BACPAC) Research Program: Structure, Research Priorities, and Methods
Source: Pain Med. 2023 Jan 9;24(Suppl 1):S3–S12. doi: 10.1093/pm/pnac202 (PMC10403298; doi:10.1093/pm/pnac202)
Supplement: pnac202_Supplementary_Data [file pnac202_supplementary_data.pdf]

Low-Back Pain Treatment Questionnaire
Assessed monthly, months 1-6

General Instructions:

This document describes a standardized approach to the assessment of treatments received during observational longitudinal studies within BACPAC. Specifically, the document specifies the categories of treatments that should be identified, the intensity and duration of surveillance for these treatments, and the timing and nature of outcome assessments that are expected for study participants who report having received or initiated one of these treatments. Standardization of these assessments will strengthen our ability to conduct cross-study analyses that assess treatment effects and phenotypic variations in treatment effects, and will be used to inform subsequent collaborative trials.

1. In the past month, have you received surgery or a surgical procedure for your low-back pain?

- Yes
- No

1a. IF 1=YES
Did you receive a spinal fusion surgery? (Spinal fusion uses metal hardware or bone grafts to permanently connect two or more vertebrae.)

- Yes
- No

2. In the past month, have you received one or more injections for your low-back pain?

- Yes
- No
- Unsure

3. In the past month, have you taken a medication for the treatment of your low-back pain?

- Yes
- No

3a: IF 3=YES
In the past month, have you taken opioids (e.g., oxycodone (Percocet), hydrocodone (Vicodin), fentanyl, tramadol (Ultram))?

- Yes
- No

3a.1: IF 3a=YES
In the past month, has your dose changed?

- Yes, this is a new prescription/medication
- Yes, the dose increased
- Yes, the dose decreased
- No, the dose has not changed

3b: IF 3=YES
In the past month, have you taken an SSRI/SNRI (e.g., paroxetine (Paxil), duloxetine (Cymbalta))?

- Yes
- No

3b.1: IF 3b=YES

In the past month, has your dose changed?

- Yes, this is a new prescription/medication
- Yes, the dose increased
- Yes, the dose decreased
- No, the dose has not changed

3c: IF 3=YES
In the past month, have you taken gabapentin (Neurontin) or pregabalin (Lyrica)?

- Yes
- No

3c.1: IF 3c=YES
In the past month, has your dose changed?

- Yes, this is a new prescription/medication
- Yes, the dose increased
- Yes, the dose decreased
- No, the dose has not changed

3d: IF 3=YES
In the past month, have you taken a Tricyclic Antidepressant (e.g., amitriptyline (Elavil), doxepin (Sinequan))?

- Yes
- No

3d.1: IF 3d=YES
In the past month, has your dose changed?

- Yes, this is a new prescription/medication
- Yes, the dose increased
- Yes, the dose decreased
- No, the dose has not changed

3e: IF 3=YES
In the past month, have you taken a Nonsteroidal Anti-inflammatory Drug (NSAID) (e.g., ibuprofen (Advil, Motrin) or naproxen (Naprosyn), meloxicam (Mobic), diclofenac (Voltaren), celecoxib (Celebrex) or others)?

- Yes
- No

3e.1: IF 3e=YES
In the past month, has your dose changed?

- Yes, this is a new prescription/medication
- Yes, the dose increased
- Yes, the dose decreased
- No, the dose has not changed

4. In the past month, have you received the following physical therapy, occupational therapy, or chiropractic treatments for your low-back pain?

- Adjustment/Manipulation
- Active physical therapy or occupational therapy (e.g., supervised exercise)
- Other passive physical therapy such as ultrasound diathermy or therapeutic massage

where you lay on a table and had a treatment administered to you
- Other
- None of the above

4a: IF 4=OTHER
What other therapy have you received in the past month?

OPEN TEXT

5. In the past month, have you participated in an exercise routine that you do on your own to manage your low-back pain? (*Note, this is unsupervised exercise like an at-home aerobics program or walking routine*)
- Yes
- No

5a. IF 5=YES
How many days in the past week did you exercise?
- Range 0-7

5b. IF 5=YES
Was this recommended or "prescribed" by a provider?
- Yes
- No

5c. If 5=YES
Was this prescribed or delivered via telehealth (by phone or video call)?
- Yes
- No

6. In the past month, have you had acupuncture treatment for your low-back pain?
- Yes
- No

7. In the past month, have you received mental health therapy or counseling to help you control or manage your low-back pain?
- Yes
- No

7a. If 7=YES
Was this prescribed or delivered via telehealth (by phone or video call)?
- Yes
- No

8. In the past month, have you used any mindfulness, meditation, or relaxation approaches to manage your low-back pain?
- Yes
- No

8a. If 8=YES
Was this prescribed or delivered via telehealth (by phone or video call)?
- Yes

- o No

9. In the past month, have you attempted a diet, nutrition change, or weight loss program?

- o Yes
- o No

9a. IF 9=YES
Was this recommended or "prescribed" by a provider?

- o Yes
- o No

9b. If 9=YES
Was this prescribed or delivered via telehealth (by phone or video call)?

- o Yes
- o No

<u>**Frequency of Assessment of Interventions**</u>
Sites will contact each patient once a month for the first 6 months following study enrollment to assess treatments received. The date on which the new treatment was initiated will be recorded.

<u>**Outcome Assessment 1 and 2 months after Initiation of New Treatment**</u>
For patients who reported receiving a new treatment, during the next two assessments (i.e., the following 1 and 2 months after the treatment is initiated), the site will also assess response to treatment using the following measures.

Primary Outcome: Patient Global Impression of Change (PGIC) (1 item)
10. *Text between parentheses should be populated with the treatments highlighted in questions 1, 2, 3, 4, 5, 6, 7, 8 and 9.*Since the start of the treatment (treatment), my overall pain is…

- o Very much improved
- o Much improved
- o Minimally improved
- o No Change
- o Minimally worse
- o Much worse
- o Very much worse

Secondary Outcome: Pain intensity (PROMIS Pain intensity and Min Data Set low-back pain specific pain intensity) (2 items)
11. In the past 7 days…

How would you rate your *low-back pain* on average?

__0 1 2 3 4 5 6 7 8 9 10
No Pain Worst imaginable pain

12. What number best describes your pain on average in the past week?

__0 1 2 3 4 5 6 7 8 9 10
No Pain Pain as bad as you can imagine

Secondary Outcome: Pain interference (PROMIS 4a) (4 items)
13. In the past 7 days…

How much did pain interfere with your day-to-day activities?

- Not at all
- A little bit
- Somewhat
- Quite a bit
- Very much

14. In the past 7 days…
How much did pain interfere with work around the home?

- Not at all
- A little bit
- Somewhat
- Quite a bit
- Very much

15. In the past 7 days…
How much did pain interfere with your ability to participate in social activities?

- Not at all
- A little bit
- Somewhat
- Quite a bit
- Very much

16. In the past 7 days…
How much did pain interfere with your household chores?

- Not at all
- A little bit
- Somewhat
- Quite a bit
- Very much
